# High-resolution laser resonances of antiprotonic helium in superfluid ^4^He

**DOI:** 10.1038/s41586-022-04440-7

**Published:** 2022-03-16

**Authors:** Anna Sótér, Hossein Aghai-Khozani, Dániel Barna, Andreas Dax, Luca Venturelli, Masaki Hori

**Affiliations:** 1 Max-Planck-Institut für Quantenoptik, Garching, Germany; 2 CERN, Geneva, Switzerland; 3 Institute for Particle and Nuclear Physics, Wigner Research Center for Physics, Budapest, Hungary; 4 Dipartimento di Ingegneria dell’Informazione, Università di Brescia, Brescia, Italy; 5 Istituto Nazionale di Fisica Nucleare, Sezione di Pavia, Pavia, Italy; 6 Fakultät für Physik, Ludwig-Maximilians-Universität München, Munich, Germany; 7 Present Address: ETH Zurich, Zurich, Switzerland; 8 Present Address: McKinsey and Company, Munich, Germany; 9 Present Address: Paul Scherrer Institut, Villigen, Switzerland

**Keywords:** Atomic and molecular collision processes, Exotic atoms and molecules, Experimental particle physics

## Abstract

When atoms are placed into liquids, their optical spectral lines corresponding to the electronic transitions are greatly broadened compared to those of single, isolated atoms. This linewidth increase can often reach a factor of more than a million, obscuring spectroscopic structures and preventing high-resolution spectroscopy, even when superfluid helium, which is the most transparent, cold and chemically inert liquid, is used as the host material^1–6^. Here we show that when an exotic helium atom with a constituent antiproton^7–9^ is embedded into superfluid helium, its visible-wavelength spectral line retains a sub-gigahertz linewidth. An abrupt reduction in the linewidth of the antiprotonic laser resonance was observed when the liquid surrounding the atom transitioned into the superfluid phase. This resolved the hyperfine structure arising from the spin–spin interaction between the electron and antiproton with a relative spectral resolution of two parts in 10^6^, even though the antiprotonic helium resided in a dense matrix of normal matter atoms. The electron shell of the antiprotonic atom retains a small radius of approximately 40 picometres during the laser excitation^7^. This implies that other helium atoms containing antinuclei, as well as negatively charged mesons and hyperons that include strange quarks formed in superfluid helium, may be studied by laser spectroscopy with a high spectral resolution, enabling the determination of the particle masses^9^. The sharp spectral lines may enable the detection of cosmic-ray antiprotons^10,11^ or searches for antideuterons^12^ that come to rest in liquid helium targets.

## Abstract (web summary)

The spectral lines of antiprotonic helium atoms are shown to retain their sub-gigahertz linewidth upon submersion in a bath of superfluid helium, enabling the hyperfine structure to be resolved.

## Main

Laser spectroscopy of antihydrogen^13,14^ and antiprotonic helium atoms ($\bar{p}{{}^{4}{\rm{H}}{\rm{e}}}^{+}\equiv {{}^{4}{\rm{H}}{\rm{e}}}^{2+}+{\bar{p}}^{-}+{e}^{-}$)^7–9^ have recently been carried out to investigate the symmetry between matter and antimatter. These experiments are complementary to some precision measurements on the properties of single antiprotons^15,16^. The high precision of these experiments could only be achieved by reducing or eliminating the collisions with normal atoms that were found to annihilate the antiprotons in the exotic atoms or perturb their atomic energy levels and strongly broaden the laser resonances^17,18^. This required forming the antihydrogen or $\bar{p}{{}^{4}{\rm{H}}{\rm{e}}}^{+}$ in magnetic traps or gaseous helium targets of extremely low atomic density *ρ* *<* 10^18^ cm^−3^, so that sharp spectral lines of effectively single isolated atoms were resolved from which the atomic transition frequencies were precisely determined. Other exotic atoms were found to accelerate and heat up during their formation, and during collisions with other molecules^19–21^ so that their X-ray spectral lines were broadened. In this work, we instead observed the surprising phenomenon wherein, in contrast to the above previous results on exotic atoms, the $\bar{p}{{}^{4}{\rm{H}}{\rm{e}}}^{+}$ embedded in superfluid helium (He II) showed visible-wavelength spectral lines that are narrower than those of many other implanted stable atoms made of normal matter reported so far. At the superfluid phase transition temperature where the atomic density of the liquid is largest, the antiproton laser resonance abruptly narrowed to sub-GHz linewidths corresponding to a relative spectral resolution of 2 × 10^−6^, which is more than a factor of 10 narrower than the same $\bar{p}{{}^{4}{\rm{H}}{\rm{e}}}^{+}$ spectra observed in supercritical-phase helium of lower density. This implies that other varieties of helium atoms containing antideuterons—or negatively charged mesons and hyperons^22^ that include the strange quark which cannot be readily decelerated and cooled using synchrotrons or isolated in ion traps—may instead be stopped in He II and measured with a high spectral resolution by laser spectroscopy. The fact that the lineshapes are so sensitive to the liquid temperature and phase suggests that $\bar{p}{{}^{4}{\rm{H}}{\rm{e}}}^{+}$ may be used to study some condensed-matter effects in superfluid helium^23–27^.

Laser spectroscopy of various atoms implanted into bulk He II began in the 1980s with the expectation that He II, being cold and inert, would constitute an ideal host material where the distortions of the atomic spectral lines would be small compared to other liquids. Measurements quickly showed, however, that the visible-wavelength spectral lines involving the outermost valance orbitals of alkaline^2,3^, alkaline earth^4,5^ and lanthanide^6^ atoms embedded in normal liquid helium (He I) and He II are nevertheless highly shifted (|Δ*ν|* ≈ 10^4^ GHz) and asymmetrically broadened (*Γ* = 10^3^–10^4^ GHz) by a factor greater than or equal to 10^6^ compared to the typical natural widths of several MHz or less. Other specific resonances involving electronic excitations from the inner shells of Tm^28^, Eu^29^, Cu, Au^30^ and Dy^31,32^ atoms in He II showed widths of *Γ* = 4–80 GHz with additional wing structures that were typically located 150–300 GHz away from the main spectral line. The linewidths of some of these resonances decreased in superfluid helium^29,32^. The broad, complex lineshapes have been interpreted by effective models, some of which involve the formation of bubble-like defects around various impurities (particularly alkaline, alkaline earth and rare earth) atoms and molecules.

**Fig. 1:**
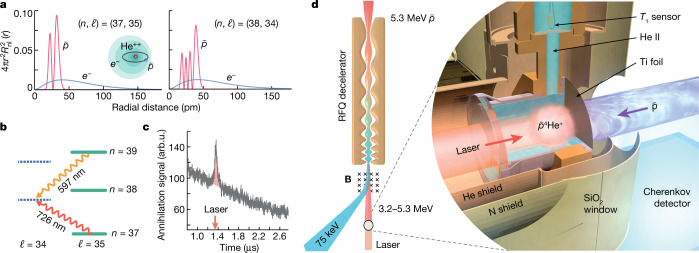
Laser spectroscopy of $\bar{p}{{}^{4}{\rm{H}}{\rm{e}}}^{+}$ synthesized in He I and He II targets. **a**, Radial distribution functions of the antiproton (red curves) and electron (blue curves) for states (*n*, *ℓ*) = (37, 35) and (38, 34) of a single isolated $\bar{p}{{}^{4}{\rm{H}}{\rm{e}}}^{+}$ atom. Here ${R}_{n\ell }(r)$ denotes the radial component of the antiproton or electron orbital at a distance *r* from the helium nucleus.  Laser transitions between the two states involve an unusually small (Δ*r*_e_ ≤ 2 pm) change in the root-mean-square radius of the 1*s* electronic orbital. **b**, Energy level diagram of $\bar{p}{{}^{4}{\rm{H}}{\rm{e}}}^{+}$ indicating the positions of the transitions (37, 35) → (38, 34) and (39, 35) → (38, 34). **c**, Signal of the Cherenkov detector, which corresponds to the time distribution of $\bar{p}{{\rm{H}}{\rm{e}}}^{+}$ annihilations. The laser resonance is induced at *t* ≈ 1.4 μs after $\bar{p}{{}^{4}{\rm{H}}{\rm{e}}}^{+}$ formation. The large number of annihilations at *t* = 0 is not shown. **d**, Experimental layout. Antiprotons emerging from a radio frequency quadrupole (RFQ) decelerator came to rest in either a gaseous or supercritical phase helium, He I, or He II target. The produced $\bar{p}{{\rm{H}}{\rm{e}}}^{+}$ atoms were irradiated with a resonant laser pulse (see text).

The neutral $\bar{p}{{}^{4}{\rm{H}}{\rm{e}}}^{+}$ studied here consists of a helium nucleus, an electron in the 1*s* ground state, and an antiproton occupying a Rydberg state of large principal *n* and orbital angular momentum *ℓ* ≈ *n* − 1 quantum numbers. The atom’s longevity in liquid helium targets has been theoretically and experimentally studied^33–35^. The *n* *>* 41 antiproton orbitals that extend outside the electron shell with a root-mean-square radius *r*_e_ ≈ 40 pm are easily destroyed in collisions with other atoms and have never been detected. By comparison, the *n* = 30–40 antiproton orbitals lie well within the electron shell (Fig. 1a) and should in principle be better protected, but numerous states were found to likewise be destroyed for atoms synthesized in gas targets of moderate density *ρ* = 10^20^–10^21^ cm^−3^ (ref. ^36^) so that laser spectroscopy of antiprotonic atoms suspended in liquid targets has not been achieved so far. In this work we nevertheless detected two transitions (*n*, *ℓ*) = (37, 35) → (38, 34) and (39, 35) → (38, 34) at the visible wavelengths *λ* = 726 nm and 597 nm, respectively, that survived in He I and He II targets. The resonance parent states (37, 35) and (39, 35) have microsecond-scale lifetimes, whereas the daughter state (38, 34) has an Auger width *Γ*_A_ ≈ 21 MHz (ref. ^7^; Fig. 1b). As the radius of a single isolated $\bar{p}{{}^{4}{\rm{H}}{\rm{e}}}^{+}$ atom is an order of magnitude smaller (Fig. 1a) than the valance orbitals of the above-mentioned impurity atoms, and the optical transitions of the massive antiproton involve remarkably small changes in the radius Δ*r*_e_ ≤ 2 pm (ref. ^7^) of the electron and the related $\bar{p}{{}^{4}{\rm{H}}{\rm{e}}}^{+}-{}^{4}{\rm{H}}{\rm{e}}$ pairwise potentials^17,18^, we may expect lineshapes that are quantitatively different from those of other many impurity atoms.

In this work the Antiproton Decelerator of CERN provided a pulsed beam containing ${N}_{\bar{p}}$ = (2–3) × 10^7^ antiprotons with an energy *E*_k_= 5.3 MeV and repetition rate *f*_r_ ≈ 8 mHz (Fig. 1d). The beam was allowed to traverse a radio frequency quadrupole (RFQ) decelerator which slowed down approximately 20% of the antiprotons to *E*_*k*_ = 75 keV. A dipole magnet diverted the slow antiprotons to another experiment. The remaining approximately 80% missed the longitudinal acceptance of the RFQ decelerator and emerged with an energy *E*_k_ = 3.2–5.3 MeV. We constructed a beamline to transport the higher-energy antiprotons to the position of the helium target and collected spectroscopic data over several months. The $\bar{p}{{}^{4}{\rm{H}}{\rm{e}}}^{+}$ were produced by allowing the antiprotons to come to rest in a 35-mm diameter, hermetically sealed chamber filled with He I or He II at temperatures between *T* = 1.49(3) K and 4.15(2) K (see Methods for a discussion on the experimental uncertainty of the target temperature and pressure). A separate target filled with helium of *T* = 5.97(6)–6.4(4) K and maximum pressure *p* = 555(2) kPa, which corresponds to the supercritical phase with approximately 70% of the He I density, was also used.

The 726-nm transition with a small dipole moment *d*_m_ = 0.018 a.u. (a.u., 1 atomic unit = 8.48 × 10^−30^ C m) was excited by irradiating the $\bar{p}{{}^{4}{\rm{H}}{\rm{e}}}^{+}$ with a 50-ns-long laser pulse of linewidth *Γ*_las_ = 60 MHz and fluence *ε* = 4 mJ cm^−2^ (Fig. 1b). For this we used a Ti:sapphire pulsed laser which was injection-seeded by a continuous-wave (CW) laser. The 597-nm resonance of *d*_m_ = 0.24 a.u. was excited by a 50-ns-long laser pulse of *Γ*_las_ = 80 MHz and *ε* = 0.12 mJ cm^−2^, which was generated by a CW pulse-amplified dye laser^9^. The optical frequencies of the seed lasers were measured with a precision of better than 1 MHz using a femtosecond frequency comb. A mechanical shutter prevented the residual seed beam from entering and heating up the target during the intervals between the antiproton arrivals. The two-body antiprotonic helium ion ($\bar{p}{{}^{4}{\rm{H}}{\rm{e}}}^{2+}\equiv \bar{p}+{{}^{4}{\rm{H}}{\rm{e}}}^{2+}$) that remained after Auger emission was rapidly destroyed by collisions with the surrounding liquid. The resulting spike (Fig. 1c) in the flux of charged pions that emerged from the antiproton annihilations was measured by an acrylic Cherenkov detector.

**Fig. 2:**
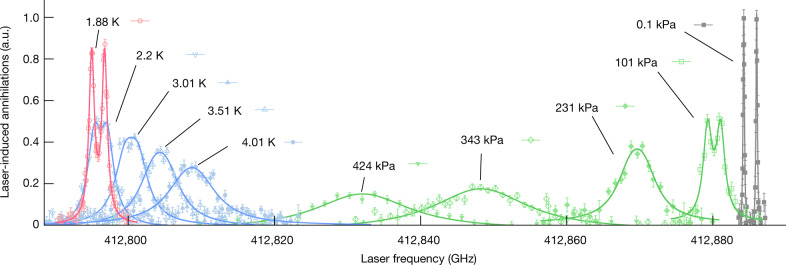
Resonance profiles of the transition (*n*, *ℓ*) = (37, 35) → (38, 34) synthesized in various targets. Spectral lines measured in gaseous and supercritical helium (green data points) at pressures *p* = 101 kPa, 231 kPa, 343 kPa and 424 kPa, and temperature *T* = 6.0–6.4 K show progressively larger collisional shifts and broadenings relative to the data^9^ measured in gas of *p* = 0.1 kPa and *T* ≈ 1.6 K (grey) with a linewidth *Γ*_L_ = 0.19(3) GHz. The best fits of four overlapping Lorentzian functions are shown superimposed on each spectrum. Spectral lines observed in He I (blue) of *T* = 4.01 K, 3.51 K, 3.01 K and 2.20 K became narrower as the liquid temperature was reduced. A rapid reduction of the linewidth below the He II transition temperature revealed the hyperfine structure, with the sharpest spectral lines observed between *T* = 1.78(2) K and 1.88(2) K (red). Error bars, 1 s.d.

Fig. 2 shows the spectral profiles of the 726-nm resonance measured at six pressures of the gaseous and supercritical helium targets, which were obtained by plotting the intensity of the induced annihilation signals as a function of the laser frequency. Each point represents data collected from 2–5 antiproton pulses. The lineshapes contain contributions from the 21-MHz natural width^7^ of the daughter state, the 60–80-MHz laser linewidth, the hyperfine structure arising from the spin–spin interaction between the electron and antiproton^37^, power broadening effects, and the complicated (see below) effects of the interactions with the surrounding helium, combined with the motions of the $\bar{p}{{\rm{H}}{\rm{e}}}^{+}$ atoms. The apparent linewidths were obtained by fitting the spectral profiles with four overlapping Lorentzian functions that were fixed to the relative positions of the calculated hyperfine intervals^37^. This simplified definition of the full width at half maximum (FWHM) Lorentzian linewidth *Γ*_L_ avoided the ambiguities that may arise from a more specific lineshape model with many parameters. The total uncertainty was taken as the quadratic sum of the statistical uncertainty arising from the finite number of excited atoms and the systematic uncertainty. The latter includes contributions from the calibration and fluctuations of the target temperature and shifts |Δ*ν*_las_ | ≤ 30–60 MHz in the laser frequency that are due to spurious modulations that occurred during the amplification of the laser pulse. The heating effect of the laser on the target was investigated using finite-element simulations.

As shown in Fig. 2, the resonance profile measured in a dilute gas target of pressure *p* = 0.1 kPa and temperature *T* ≈ 1.6 K (ref. ^9^) resolves the hyperfine structure as two distinct peaks. The best fit of four overlapping Lorentzian functions is indicated by the grey curve with a linewidth of *Γ*_L_ ≈ 0.19(3) GHz. When the pressure was increased by three orders of magnitude to *p* = 101.4(1.4) kPa at *T* = 6.4(4) K, collisions shifted the resonance centroid by Δ*ν* = −4.94(5) GHz and broadened the linewidth to *Γ*_L_ ≈ 1.24(11) GHz, as expected. With a further, twofold increase of the target pressure to *p* = 231.1(1.4) kPa, the broadening became so great that the hyperfine structure could no longer be resolved (green curves). When the helium transitioned to the supercritical phase upon increasing the pressure between *p* = 343(2) kPa and 555(2) kPa (see Extended Data Table 1), the resonance further shifted from Δ*ν* = −36.7(4) GHz to −64.4(4) GHz and broadened to such a degree *Γ*_L_ = 15(2) GHz that the spectral line could no longer be resolved with a high signal-to-noise ratio. We used laser beams of fluence *ε* = 8–9 mJ cm^−2^ to compensate, and normalized the spectra of Fig. 2 to correct for the corresponding increase in the laser power.

**Fig. 3:**
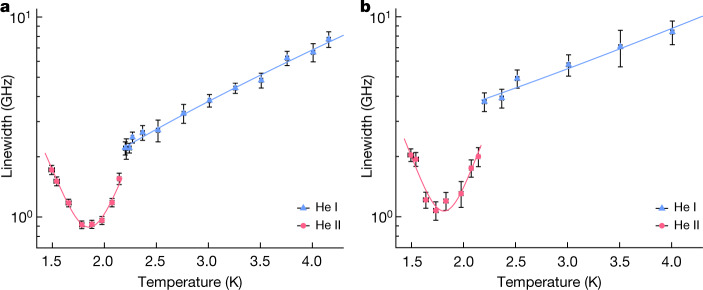
Linewidths of the spectral profiles of two $\bar{p}{{}^{4}{\rm{H}}{\rm{e}}}^{+}$ laser transitions as a function of the He I or He II target temperature. **a**, **b**, The FWHM Lorentzian linewidths of the transition (*n*, *ℓ*) = (37, 35) → (38, 34) (**a**) and (*n*, *ℓ*) = (39, 35) → (38, 34) (**b**). In normal liquid helium the temperature dependence of the linewidth was well approximated by a single exponential (indicated by the blue curve), whereas in superfluid helium a characteristic dependence with the smallest linewidths at temperatures between *T* = 1.7 K and 1.9 K was observed. Error bars, 1 s.d.

In liquid helium, however, the spectral lines became far narrower despite the higher atomic density (Fig. 2). As the He I target was cooled from *T* = 4.157(15) K to 2.201(17) K, the apparent linewidth rapidly decreased from *Γ*_L_ = 7.7(7) GHz to 2.21(17) GHz (Fig. 3a). A similar reduction from *Γ*_L_ = 8.4(1.1) GHz to 3.8(4) GHz was observed for the 597-nm resonance (Fig. 3b). This temperature dependence of *Γ*_L_ could be approximated by a single exponential (blue lines); the best fit yielded reduced *χ*^2^ values of approximately 0.3 and 0.4 for the 726-nm and 597-nm resonances, respectively.

An abrupt further narrowing of the linewidth was observed below the He II phase transition temperature *T*_*λ*_ = 2.18 K (Fig. 3a), which unexpectedly revealed the hyperfine structure (Fig. 2). The highest resolution of *Γ*_L_ = 0.91(4)–0.92(4) GHz for the 726-nm resonance was observed at *T* = 1.78(2)–1.88(2) K, whereas the 597-nm resonance narrowed to *Γ*_L_ = 1.07(11) GHz at *T* = 1.73(2) K (Fig. 3b). At a slightly lower temperature *T* = 1.49(3) K the linewidths increased by factor of approximately 2 to *Γ*_L_ = 1.72(9) GHz and 2.04(15) GHz, respectively. This resulted in the characteristic temperature dependencies shown in Fig. 3a, b, which were fitted with parabolas. The spectral resolutions are an order of magnitude higher than the limits that would be expected according to the predictions of ab initio theoretical calculations for atoms of temperature *T* ≈ 5.4 K using the impact approximation of binary collisions^17,18^, which were based on pairwise potentials derived from the highly precise wavefunctions of $\bar{p}{{}^{4}{\rm{H}}{\rm{e}}}^{+}$ (refs. ^7,38^). Taken together with the abrupt narrowing observed at *T*_*λ*_, this implies that collective effects in the superfluid narrow the laser resonances below the limit expected from simple binary atomic collisions.

**Fig. 4:**
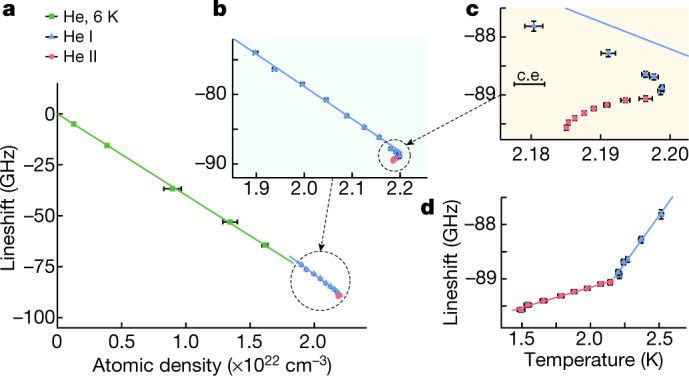
Collisional shifts of the resonance frequency of the transition (*n*, *ℓ*) = (37, 35) → (38, 34) observed in gas, supercritical phase, He I and He II targets. **a**, Shifts in gaseous and supercritical helium (indicated by green squares), He I (blue triangles) and He II (red circles) targets relative to the zero-density transition frequency of *ν*_0_ = 412,885.133(1) GHz (ref. ^9^) as a function of the atomic density. **b**, **c**, Magnified views of **a**, showing a deviation from a linear density dependence at target temperature * T* < 2.5 K. The results of the best fits of linear functions on the gaseous and supercritical helium (green line) and He I (blue line) data at *T* ≥ 2.7 K are shown superimposed. The size of the correlated uncertainty arising from the conversion of the liquid helium pressure and temperature into atomic density based on the International Temperature Scale of 1990 is indicated separately by the single error bar labelled as c.e. (see Methods). **d**, The temperature dependence shows an abrupt change in the gradient d*ν*/d*T* at the He II transition temperature *T*_*λ*_ = 2.18 K. Error bars, 1 s.d.

**Extended Data Fig. 1:**
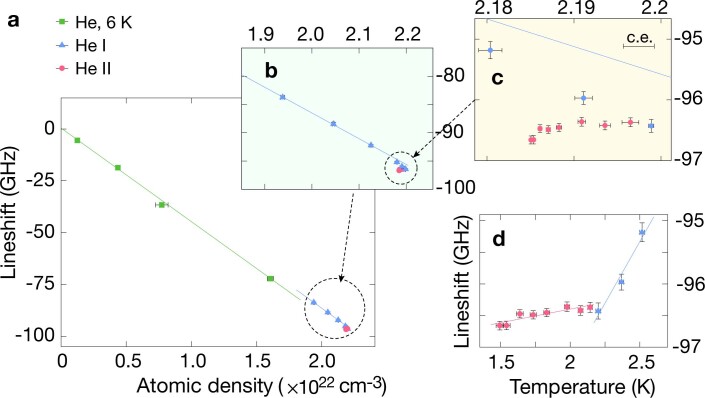
Collisional shifts of the resonance frequency of the transition (*n*, *ℓ*) = (39, 35) → (38, 34) observed in gas and supercritical phase helium, He I and He II targets. **a**, Shifts in gaseous and supercritical helium (indicated by green square markers), He I (blue triangles), and He II (red circles) targets relative to the zero-density transition frequency of *ν*_0_ = 501,948.753(2) GHz^9^ as a function of the atomic density. **b,c** Magnified views of a, showing a deviation from a linear density dependence at target termperature *T* < 2.5 K. The results of the best fits of linear functions on the gaseous and supercritical helium (green line) and He I (blue line) data at *T* ≥ 2.7 K are shown superimposed. The size of the correlated uncertainty arising from the conversion of the liquid helium pressure and temperature into atomic density based on the International Temperature Scale of 1990 is indicated separately by the error bar labelled as c.e. (see text). **d**, The temperature dependence shows an abrupt change in the gradient d*ν*/d*T* at the He II transition temperature *T*_*λ*_ = 2.18 K. Error bars, 1 s.d.

In gaseous and supercritical helium, the centroids of the two resonances shifted linearly with target density relative to the zero-density frequencies^9^ (Fig. 4a and Extended Data Fig. 1a) with gradients d*ν*/d*ρ* ≈ −(4.0–4.5) × 10^−21^ GHz cm^3^. This agrees with the results of previous experiments that were carried out in gas targets of much lower density^36^, and with the predictions^17,18^ of the above binary collision calculations (Table 1). Similar gradients were observed in He I of temperatures between *T* = 4.2 K and 2.8 K. When cooled below 2.5 K, however, the gradients began to increase in a nonlinear way relative to density, before abruptly changing sign at the He II transition temperature *T*_*λ*_ (Figs. 4b, c and Extended Data Fig. 1b, c). The temperature dependence of the frequency shifts likewise appear to be roughly linear between *T* = 2.2 K and 2.55 K (Fig. 4d and Extended Data Fig. 1d) with gradients d*ν*/d*T* = (3.5–3.9) GHz K^−1^, but in He II the gradients abruptly decreased by a factor of 5–10 (Table 1). The onset of superfluidity thus affects both the shift and linewidth of the atomic resonances.

**Table 1:** Gradients of the collisional shifts of the laser resonance frequencies of $\bar{{\boldsymbol{p}}}{}^{{\bf{4}}}{\bf{H}}{{\bf{e}}}^{+}$ against target density (d*ν*/d*ρ*) and temperature (d*ν*/d*T*)

Transition	d*ν*/d*ρ* (×10^−21^ GHz cm^3^)			d*ν*/d*T* (GHz K^−1^)	
(*n*,* ℓ*) → (*n*′,* ℓ*′)	Gas, supercritical	He I	Theory	He I	He II
(37, 35) → (38, 34)	−3.99(7)	−4.67(9)	−3.42	3.5(3)	0.75(7)
(39, 35) → (38, 34)	−4.52(8)	−4.5(2)	−4.05	3.9(7)	0.4(1)

Experimental d*ν*/d*ρ* values measured in gaseous and supercritical helium, and He I targets at temperatures between *T* = 2.7 K and 4.2 K are compared with theoretical values^18^ calculated for binary collisions at *T* = 5.4 K. The d*ν*/d*T* value in He I represents the best fit to data measured at temperatures between *T* = 2.2 K and 2.55 K. In He II targets, the d*ν*/d*T* value decreased by a factor of 5–10 (see text).

The spectral lineshapes of other neutral atoms implanted into He I and He II have been interpreted by simplified effective models in which these impurities reside in bubble-like defects^1–6^ of 1-nm-scale diameters *d*_b_. The Franck–Condon principle implies that the excitation of the D1 transition of an impurity Cs atom, for example, occurs within a fixed configuration of the defect. This is rapidly followed by a more than Δ*d*_b_ = 200 pm expansion of the bubble diameter and some vibrations that occur on the surface of the bubble with a total energy *E*_b_ ≈ 20 meV (refs. ^3,30^). These vibrations are subsequently damped as some of the characteristic frequencies *ν*_b_ = 100–500 GHz coincide with those of elementary roton (*ν*_r_ ≈ 180 GHz) and maxon (*ν*_m_ ≈ 290 GHz) excitations of He II. Because approximately 10 phonons with millielectronvolt energies are emitted into the surrounding liquid, a continuous optical spectrum of width *Γ* = 10^3^–10^4^ GHz is observed. By comparison, laser transitions involving the inner electron orbitals of Tm, Eu, Cu or Au atoms cause smaller expansions in *d*_b_, and so the resulting spectra contain sharp ‘zero phonon lines’ and relatively broad ‘phonon wings’ corresponding to zero and 1–2 phonon emissions, respectively^29–31,39^. The microscopic structure of bulk He II that causes these effects is not fully understood. Neutron diffraction experiments indicate that less than 10% of the ^4^He atoms occupy the ground state that corresponds to a Bose–Einstein condensate in a temperature-dependent way^23–26^, whereas the remainder occupy excited states that allow the phonons and rotons to propagate and interact with the impurities. For SF_6_ (ref. ^40^) and OCS molecules^41^ attached to He II clusters cooled to *T* *<* 400 mK, sharp rotational resonances at infrared wavelengths *λ* ≈ 10.6 μm and 4.85 μm have been observed. This implies that the molecules can rotate relatively freely in the superfluidity that arises within the small (10^2^–10^4^) number of ^4^He atoms that comprise these clusters, though the moment of inertia of the corresponding transitions appear increased by factor 2.7–2.8 compared to a single isolated molecule. Compared to the visible-wavelength spectral lines of many other atoms and molecules embedded in He II, the antiprotonic E1 resonances of $\bar{p}{{}^{4}{\rm{H}}{\rm{e}}}^{+}$ were distinguished by symmetrical and sharp lines that abruptly became narrow at temperatures below *T*_*λ*_. This is believed to arise from the fact that the antiprotonic atom retains a radius *r*_e_ ≈ 40 pm of the electron shell during the laser excitation^7^ which is an order of magnitude smaller than many other normal neutral atoms. The perturbations to the surrounding superfluid helium as evidenced by the calculated $\bar{p}{{}^{4}{\rm{H}}{\rm{e}}}^{+}-{}^{4}{\rm{H}}{\rm{e}}$ pairwise potentials are so small that the spectral lineshapes become sensitive to minute effects, including the abrupt changes in the number densities of elementary excitations that occur during the superfluid phase transition.

A broad laser resonance of linewidth *Γ* ≈ 100 GHz corresponding to a relative resolution *Γ*/*ν* ≈ 6 × 10^−4^ was recently observed in pionic helium (*π*^4^He^+^ ≡ ^4^He^2+^ + *e*^−^ + *π*^−^) atoms that were formed in He II (ref. ^42^). This linewidth mostly arises from the picosecond-scale Auger lifetime of the resonance daughter state. The present work implies that other spectral lines involving *π*^4^He^+^ states with longer lifetimes may in principle reach much higher resolutions of approximately 10^−6^. Other three-body exotic helium atoms such as neutral kaonic helium (*K*^4^He^+^ ≡ ^4^He^2+^ + *e*^−^ + *K*^−^) with a mean lifetime of *τ* = 10 ns, andantideuteronic helium($\bar{d}{{}^{4}{\rm{H}}{\rm{e}}}^{+}\equiv {{}^{4}{\rm{H}}{\rm{e}}}^{2+}+{e}^{-}+\bar{d}$) with a microsecond-scale lifetime are expected to have atomic structures and diameters that are comparable to $\bar{p}{{}^{4}{\rm{H}}{\rm{e}}}^{+}$. Collisional shifts of the transition frequencies may limit the precision of future laser spectroscopy experiments on such atoms, but the shifts can be extrapolated to zero density as shown in Fig. 4.

Any anomalous excess in the flux of antiprotons^10,11^ or antideuterons^12,43^ of low kinetic energy *E* ≤ 200 MeV in cosmic rays is predicted to constitute an important indication of the possible dark matter that decay or annihilate in the halo of the Milky Way^44,45^, or primordial black hole evaporation by Hawking radiation^46–48^. The 30–300 litres of coolant He I or He II stored in some satellites over many months^49,50^, or alternatively in high-altitude balloons, correspond to an effective target thickness in which *E* ≤ 100 MeV antiprotons readily come to rest and form $\bar{p}{{}^{4}{\rm{H}}{\rm{e}}}^{+}$. Some 3% of the antiprotons are captured into long-lived states of $\bar{p}{{}^{4}{\rm{H}}{\rm{e}}}^{+}$ that are formed in gaseous and liquid helium targets^35,51^. On the basis of the results of the experiment presented here, we estimate that the sharp spectral lines of these $\bar{p}{{}^{4}{\rm{H}}{\rm{e}}}^{+}$ may be detected with an efficiency of approximately 10^−3^ per stopped antiproton using laser spectroscopy, depending on the transitions that may be simultaneously interrogated.

## Methods

### Gaseous and supercritical phase helium target

The measurements involving the gaseous and supercritical phase helium targets (Fig. 1d) were carried out using a 35-mm-diameter chamber made of stainless steel that was designed to withstand inner pressures *p* *>* 1 MPa at a temperature *T* ≈ 4–6 K. The antiprotons entered through a *t*_r_ = 75-μm-thick window made of annealed titanium that was attached to one side of the chamber by vacuum brazing, the laser beam through a 28-mm-diameter, 5-mm-thick UV-grade sapphire window affixed on the opposite side. The chamber was mounted on a liquid helium constant-flow cryostat. The cryostat was shielded from external thermal radiation by two layers of Cu enclosures, which were each cooled by either the vapour of the coolant helium or with liquid nitrogen.

The target pressure *p* was taken as the average between the readings of two capacitive manometers of precision *ϵ*_prec_ = 1.4–2.0 kPa that were in pressure equilibrium with the cryogenic target gas. During the measurement runs that each lasted 8–14 h, drifts of *ϵ*_fluc_ = 0.2 kPa were observed when the target pressure was adjusted to *p* ≈ 100 kPa. Larger fluctuations *ϵ*_fluc_ = 1 kPa were seen at a higher target pressure *p* ≈ 560 kPa. The total uncertainty *ϵ*_*p*_ = 1.4–2.2 kPa on the target pressure was taken as the quadratic sum of *ϵ*_fluc_ and *ϵ*_prec_.

A carbon-ceramic sensor^52^ was mounted on the outer wall of the stainless steel chamber which had a specified precision *ε*_c_ = 10 mK. Its reading was stabilized to a value *T*_2_ = 6.30 K by regulating the current applied to a nichrome ribbon heater mounted on the heat exchanger of the cryostat using a proportional–integral–derivative (PID) controller. The temperature of the target gaseous or supercritical helium was measured by a second sensor of reading *T*_1_ which was suspended in the target helium. This *T*_1_ reading fluctuated by *ε*_fluc_ = 10–20 mK during the experimental runs. Both sensors were excited by currents *I* = 10 μA with a precision of ≤10 nA.

We calibrated the *T*_1_ sensor reading at nine target conditions of the liquid sealed in the chamber at temperatures between *T*_1_ = 3.04 K and 4.53 K by measuring the corresponding He I saturated vapour pressures between *p* = 34.9(1.4) kPa and 142.2(1.4) kPa using the two manometers. At vapour pressures *p* *>* 120 kPa the *T*_1_ reading deviated by less than 30 mK from the expected temperatures *T*(*p*) which were calculated using the programme HEPAK^53^. The programme was based on the parameterized state equations of helium according to the International Temperature Scale of 1990 (ITS-90)^54^. This value *ε*_prec_ = 30 mK was taken to be the uncertainty of measuring the gaseous or supercritical helium temperature at the position of the sensor. Deviations between the readings of sensors *T*_1_ and *T*_2_ arose owing to the differences in the thermal conductivities of the stainless steel chamber and the gaseous or supercritical helium. From this we estimated that the upper limit of the thermal gradient in the portion of the helium target where $\bar{p}{{}^{4}{\rm{H}}{\rm{e}}}^{+}$ were produced varied between *ε*_grad_ = 15 mK and 400 mK depending on the target pressure.  The uncertainty became particularly large (*ε*_grad_ = 400 mK) at the lowest pressure *p* ≈ 101 kPa used in this experiment. The total uncertainty *ε*_*T*_ = 40 – 400 mK of the temperature *T* of the gaseous and supercritical phase targets was taken to be the quadratic sum of the uncertainties *ε*_*c*_ , *ε*_prec_, *ε*_fluc_, and *ε*_grad_. The target pressure and temperature were converted to atomic density *ρ* with an average uncertainty of 0.1% and a maximum uncertainty of 0.5%^54,55^ using the HEPAK programme^53^.

### Thermometry of He I and He II target

The He I and He II target was a diameter *d* = 35 mm chamber made of oxygen-free high conductivity (OFHC) copper. The antiprotons entered through a *t*_r_ = 50-μm-thick Ti window, whereas the laser beam entered through a 35-mm-diameter fused silica window that was vacuum-brazed on the chamber. The chamber was mounted on the above constant-flow cryostat, and coolant liquid helium was circulated and evaporated by mechanical booster and rotary vane pumps with pumping speeds of 500 m^3^ h^−1^ and 200 m^3^ h^−1^, respectively. A carbon-ceramic sensor^52^ was suspended in the target liquid helium (Fig. 1d) which was isolated from the coolant liquid, and excited with a current *I* = 10 μA. During the spectroscopy experiments involving the He I target, the sensor readout was stabilized within *ε*_fluc_ = 2–11 mK by either regulating the current applied to a nichrome ribbon heater mounted on the heat exchanger, or by adjusting the flow conductance through a butterfly throttle valve placed upstream of the pumps with a PID controller. The readout fluctuation measured in the He II target was *ε*_fluc_ = 2–5 mK.

The 7th-degree polynomial calibration function used to convert the sensor resistance to temperature was obtained from the best fit on calibration data measured at 24 temperatures between *T*_1_ = 1.5 K and 297 K some 36 months prior to the $\bar{p}{{\rm{H}}{\rm{e}}}^{+}$ experiments. Every ~24 h during the laser spectroscopy measurements, the sensor reading *T*_1_ was calibrated at the vapour–He I–He II triple point which was taken to be *T*_*λ*_ = 2.1768 K (ref. ^56^). A second sensor of identical design, which was calibrated 3–4 months prior to the experiment, was placed at roughly the same position in the target liquid. As its reading *T*_2_ was within 3 mK of the literature value of *T*_*λ*_, we used *T*_2_ to calibrate the *T*_1_ reading. In some of the calibration measurements, we stabilized the target temperature and pressure on the vapour saturation line of helium. The sensor readings were found to agree with the expected literature values within the *ε*_c_ = 10 mK precision specified by the manufacturer and the uncertainty of the measured pressure. The temperature-dependent part of the uncertainty related to this calibration procedure involving the two sensors varied between *ε*_a_ = 4 mK at a target temperature $T=4.16\,{\rm{K}}\,\mathrm{and}\,{\varepsilon }_{a}=24\,{\rm{m}}{\rm{K}}\,{\rm{a}}{\rm{t}}\,T=1.49\,{\rm{K}}$.

The total uncertainty *ε*_T_ = 12–27 mK of the liquid target temperature *T* was obtained as the quadratic sum of the above uncertainties *ε*_a_, *ε*_c_, *ε*_fluc_ and *ε*_heat_. Here *ε*_heat_ = 5 mK denotes the maximum temperature gradient that may arise owing to the heating effect of the laser in the portion of the target volume where antiprotons came to rest (see below). The calibrated temperatures and uncertainties are provided in Extended Data Tables 1, 2.

### Heating of He I and He II target by laser beam

**Extended Data Fig. 2:**
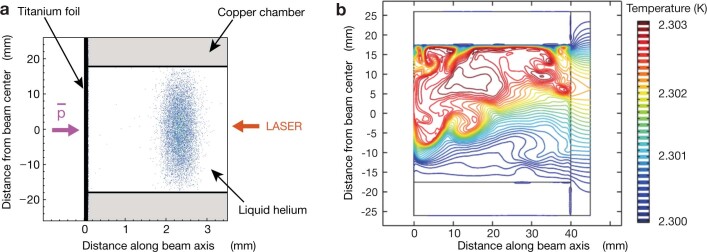
Simulated spatial distributions of antiprotons and the temperature in the He I target. **a**, Spatial distribution of antiprotons that come to rest in the He I target calculated by a GEANT4 Monte Carlo simulation (not to scale). **b**, Spatial distribution of temperature in the He I target prior to the arrival of the laser pulse, simulated by COMSOL. In the portion of the target where the $\bar{p}{{\rm{H}}{\rm{e}}}^{+}$ atoms were formed, the laser pulse arriving 119 s earlier is estimated to increase the temperature of the He I by not more than 4 mK.

The pair of sensors suspended in the He I and He II target detected no substantial temperature excursions that coincided with the single laser pulse that arrived every 110–120 s. In Extended Data Fig. 2a the spatial distribution of antiprotons that came to rest in the He II target calculated by a Monte Carlo simulation based on the GEANT4 package^57^ is shown. The emittance and energy distributions of the antiproton beam emerging from the RFQ decelerator was estimated by a particle-tracing programme^58^ and used in the simulation. The $\bar{p}{{}^{4}{\rm{H}}{\rm{e}}}^{+}$ atoms were found to form within a volume located 2–3 mm away from the Ti window.

We then carried out finite-element simulations based on the COMSOL package^59,60^ to estimate the upper limits of the thermal gradients that arose in the He I target owing to the heating of the laser beam. We chose to simulate He I of temperature *T* = 2.3 K which has a particularly small specific heat, conductivity and viscosity^53^. The dominant heat transport process in the He I was due to convection, and so an infinite-plate approximation including the effects of gravity and temperature-dependent density was used to simulate the laminar convective flows.

In the simulation, a 70-ns-long laser pulse deposited 70% of its *E* = 10 mJ energy into the entrance foil. Some ~0.8 s prior to the arrival of the antiprotons, a mechanical shutter opened and allowed part of the CW seed laser beam of power *I* = 200 mW to enter the target. The actual intensity of the CW laser beam during the experiment is estimated to be less than 10% of this value. The shutter was closed some 1 s after antiproton arrival, and was not reopened until the next arrival 119 s later. Small integration steps in time Δ*t* = 0.01 s were used when the lasers were either turned on or off. Meshes with cell sizes of less than 50 μm were used to calculate the flow and heat transport of the He I located near the Ti window.

The simulations showed that the largest heating occurred in a thin layer of He I that was located within a distance of *d* = 0.7 mm from the entrance foil. Less than 1% of the $\bar{p}{{}^{4}{\rm{H}}{\rm{e}}}^{+}$ were estimated to form in this region, whereas the remaining atoms were distributed in areas with much less heating. The $\bar{p}{{\rm{H}}{\rm{e}}}^{+}$ with microsecond-scale lifetimes were destroyed well before the volume that includes the atoms could be considerably heated; the residual temperature excursion when the next antiproton pulse arrived at the target was Δ*T* < 4 mK (Extended Data Fig. 2b).

### Particle detectors

A 300 × 100 × 20 mm^3^ Cherenkov detector made of UV-transparent acrylic was mounted near the cryostat so that it covered a solid angle of ~1.6π steradian seen from the experimental target (Fig. 1d). The charged particles (mostly pions) that emerged from the antiproton annihilations in the target traversed the detector. The resulting flash of Cherenkov light was detected by a gateable fine-mesh photomultiplier^61^ with a 17.5-mm-diameter bialkali photocathode and high dynamic range. The waveform of this signal was recorded by a digital oscilloscope of vertical resolution 8 bits, analogue bandwidth *f*_b_ = 4 GHz, and digital sampling rate *f*_s_ = 5 gigasamples per s.

The horizontal and vertical spatial profiles of the antiproton beam were measured by a pair of beam profile monitors^62^ which were positioned between the RFQ decelerator and target. A small fraction of the beam was intercepted by a grid of 20-μm-diameter tungsten–rhenium wires that were plated with gold. The resulting secondary electron emission was measured by charge-sensitive preamplifiers. Pairs of dipole magnets were tuned to carefully steer the antiprotons into the experimental target.

### Laser systems

The 726-nm laser pulses were generated by an injection-seeded Ti:sapphire laser^63^. The system was based on a CW Ti:sapphire ring laser that was pumped by the second harmonic of a single-mode Nd:YVO_4_ laser. The optical frequency of the CW laser was stabilized against a Ti:sapphire femtosecond frequency comb with a precision of better than 1 MHz (ref. ^64^). The CW seed beam was injected into a triangular Ti:sapphire cavity of 0.8-m circumference, which was pumped by a Q-switched Nd:YAG laser to produce 40–50-ns-long laser pulses of energy *E* ≈ 10 mJ. The laser pulses of wavelengths *λ* = 842 nm and 471 nm which were used to search for the resonances (*n*, *ℓ*) = (38, 35) → (39, 34) and (37, 34) → (36, 33), respectively, were also generated by this laser system. No statistically significant signal corresponding to the two transitions were observed for $\bar{p}{{}^{4}{\rm{H}}{\rm{e}}}^{+}$ produced in He I targets, presumably owing to the high rate of collisions in He I that destroyed the antiproton populations in the related states.

The 597-nm laser pulses were generated by a CW pulse-amplified dye laser. A ring CW dye laser utilizing a rhodamine 6G dye solution dissolved in ethylene glycol was pumped by an argon ion laser. This seed beam was then amplified to *E* = 2–3 mJ in an 18-mm-long rectangular dye cell, followed by a 30-mm-long Bethune dye cell filled with rhodamine B dye dissolved in methanol. The cells were pumped from the transverse direction by a Q-switched, single-longitudinal-mode Nd:YAG laser^9^ of *E* = 180 mJ. The frequency modulation induced in the optical frequencies of the Ti:sapphire and dye lasers during pulse amplification was not more than |Δ*ν*_las_ |= 30 MHz^63^ and 60 MHz, respectively.

The laser beams were expanded by a telescope and collimated by a pair of 25-mm-diameter irises placed at a distance of about 3 m from each other. The laser fluence of the 25-mm-diameter laser beam was measured behind the downstream iris.

### Resonance spectra

**Extended Data Fig. 3:**
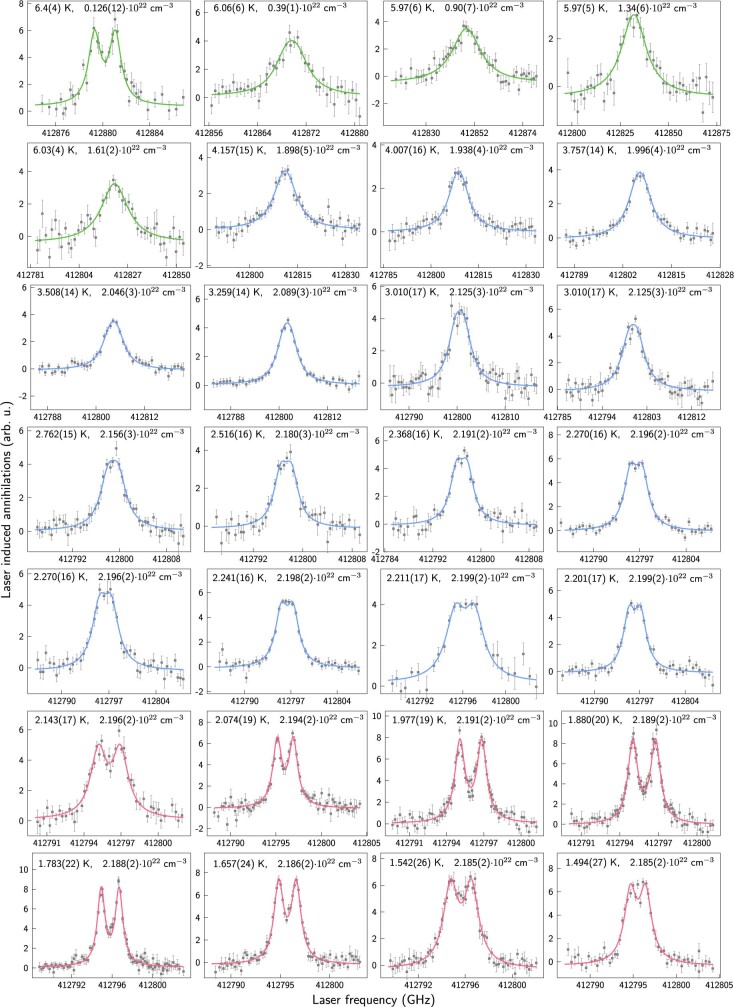
Resonance profiles of the (*n*, *ℓ*) = (37, 35) → (38, 34) transition of wavelength *λ* = 726 nm as a function of the optical frequency of the laser observed in gas and supercritical phase helium, He I and He II targets. The temperature and atomic density of the helium target that was used in each measurement are indicated (see text). Error bars, 1 s.d.

**Extended Data Fig. 4:**
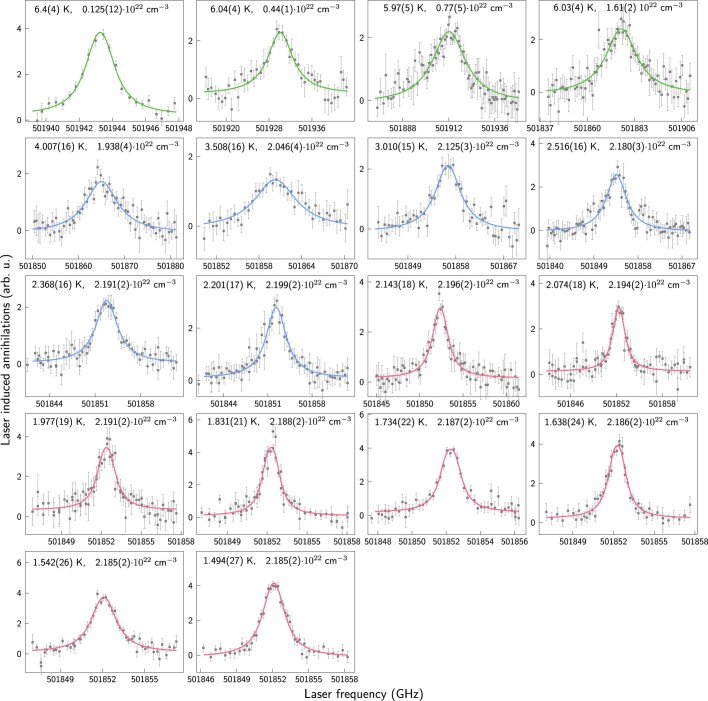
Resonance profiles of the (*n*, *ℓ*) = (39, 35) → (38, 34) transition of wavelength *λ* = 597 nm as a function of the optical frequency of the laser observed in gas and supercritical phase helium, He I and He II targets. The temperature and atomic density of the helium target used in each measurement are indicated (see text). Error bars, 1 s.d.

The resonance profiles of the transition (*n*, *ℓ*) = (37, 35) → (38, 34) at a wavelength *λ* = 726 nm measured in gaseous and supercritical helium, He I, and He II targets are shown in Extended Data Fig. 3. The temperature and pressure of the target used in each measurement are indicated in the corresponding plot. Each data point in the spectra represents data collected from 2–5 antiproton pulses. The best fit of four overlapping Lorentzian functions are shown superimposed in the spectra measured in gaseous or supercritical helium (indicated using green curves), He I (blue curves) or He II (red curves) targets. The relative positions of the Lorentzian functions Δ*ν*_HFS_ = −0.9070 GHz, −0.8808 GHz, 0.8690 GHz and 0.8783 GHz were fixed to the theoretical values corresponding to the hyperfine intervals^37^, whereas the antiproton populations were assumed to be equally distributed among the magnetic sublevels. The corresponding profiles of the (39, 35) → (38, 34) resonance at *λ* = 597 nm are shown in Extended Data Fig. 4 with the hyperfine intervals fixed to Δ*ν*_HFS_ = −0.2795 GHz, −0.2386 GHz, 0.2409 GHz and 0.2546 GHz^37^.

The full width at half maximum (FWHM) Lorentzian widths *Γ*_L_ obtained from these fits (see Extended Data Tables 1, 2) avoided the ambiguities that may arise from a more specific and complicated lineshape model with numerous parameters. The total experimental uncertainty denoted as ‘(total)’ in Extended Data Tables 1, 2 is the quadratic sum of the statistical uncertainty ‘(stat)’ arising from the finite number of excited atoms in the experimental target that were detected by the Cherenkov detector, and systematic uncertainties. The systematic uncertainty is taken as the quadratic sum of the uncertainty ‘(fluc)’ that arose from fluctuations of the target temperature, and shifts ‘(las)’ of up to 30 or 60 MHz in the laser frequency that are due to spurious modulations that were induced during the pulsed amplification in the Ti:sapphire and dye lasers, respectively.

The spin-averaged transition frequencies that were determined from the best fit of the above four overlapping Lorentzian functions are shown in Extended Data Tables 1, 2. The systematic uncertainty ‘(fit)’ related to the selection of this simplified fit function was estimated by fitting the spectra with an alternative and more complicated model involving four overlapping Voigt functions that were fixed to the relative positions of the above hyperfine intervals. The Gaussian width of the Voigt function was varied between zero and the Doppler width arising from the Maxwellian thermal motions of the atoms that corresponded to the target temperature *T*. The maximum difference of the centroid frequencies determined by the Lorentzian and Voigt functions were taken as the systematic uncertainty, ‘(fit)’. The total uncertainty ‘(total)‘ on the transition frequency was then taken as the quadratic sum of the statistical uncertainty ‘(stat)‘ and the systematic uncertainties that include the contribution from ‘(fit)‘ and the above laser modulation ‘(las)‘ = 30 or 60 MHz.

## Online content

Any methods, additional references, Nature Research reporting summaries, source data, extended data, supplementary information, acknowledgements, peer review information; details of author contributions and competing interests; and statements of data and code availability are available at 10.1038/s41586-022-04440-7.

## Figures and Tables

**Extended Data Table 1:**
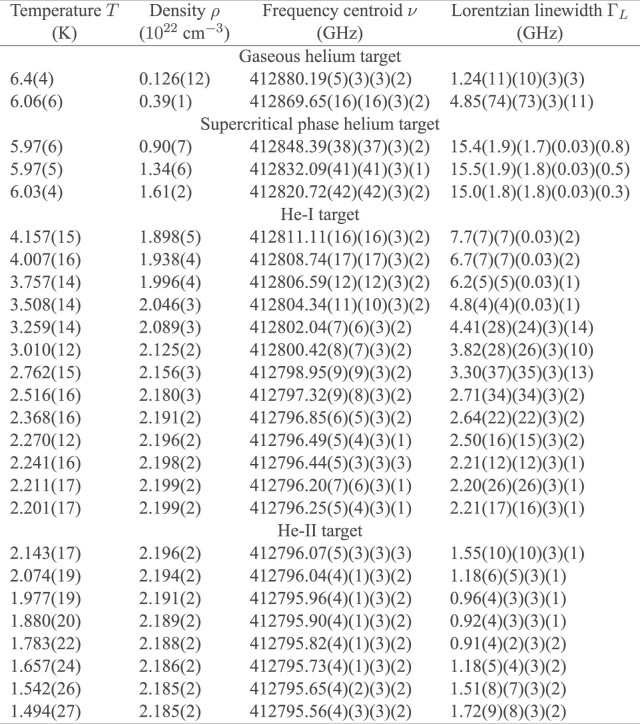
Frequency centroids and FWHM Lorentzian linewidths of the (*n*, *ℓ*) = (37, 35) → (38, 34) resonance of wavelength *λ* = 726 nm at various target phases and densities

Values in parentheses for the frequency centroid *ν* show the 1 s.d. total, statistical, laser-related and fit uncertainties (total)(stat)(las)(fit). Values in parentheses for the Lorentzian linewidth *Γ*_L_ show the 1 s.d. total, statistical, laser-related and temperature fluctuation uncertainties (total)(stat)(las)(fluc); see text. The zero-density transition frequency was previously determined as *ν*_0_ = 412,885.133(1) GHz ^9^.

**Extended Data Table 2:**
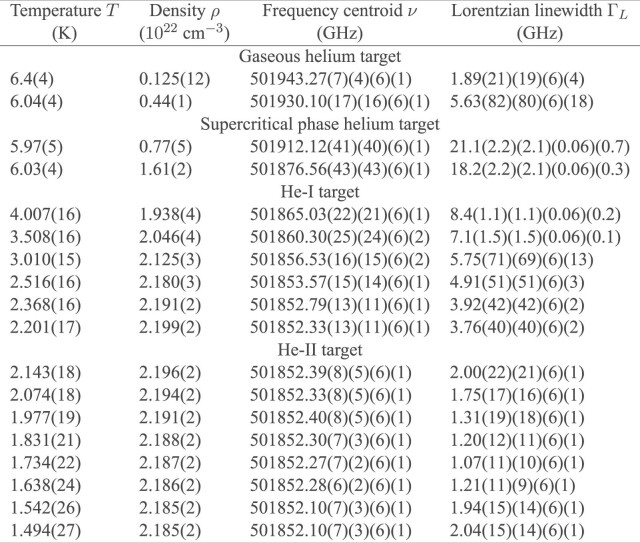
Frequency centroids and FWHM Lorentzian linewidths of the (*n*, *ℓ*) = (39, 35) → (38, 34) resonance of wavelength *λ* = 597 nm at various target phases and densities

Values in parentheses for the frequency centroid *ν* show the 1 s.d. total, statistical, laser-related and fit uncertainties (total)(stat)(las)(fit). Values in parentheses for the Lorentzian linewidth *Γ*_L_ show the 1 s.d. total, statistical, laser-related and temperature fluctuation uncertainties (total)(stat)(las)(fluc); see text. The zero-density transition frequency was previously determined as *ν*_0_ = 501,948.753(2) GHz ^9^.

## Data Availability

All data are available from the corresponding author on reasonable request.

## References

[1] Toennies JP, Vilesov AF (1998). Spectroscopy of atoms and molecules in liquid helium. Annu. Rev. Phys. Chem..

[2] Takahashi Y, Sano K, Kinoshita T, Yabuzaki T (1993). Spectroscopy of alkali atoms and molecules in superfluid helium. Phys. Rev. Lett..

[3] Kinoshita T, Fukuda K, Takahashi Y, Yabuzaki T (1995). Optical properties of alkali-metal atoms in pressurized liquid helium. Phys. Rev. A.

[4] Bauer H (1990). Laser spectroscopy of alkaline earth atoms in He II. Phys. Lett. A.

[5] Kanorsky SI, Arndt M, Dziewior R, Weis A, Hänsch TW (1994). Optical spectroscopy of atoms trapped in solid helium. Phys. Rev. B.

[6] Hui Q, Persson JL, Beijersbergen JHM, Takami M (1995). Spectroscopy and dynamics of neutral atoms in superfluid helium. Z. Phys. B.

[7] Korobov VI, Hilico L, Karr J-P (2014). *mα*^7^-order corrections in the hydrogen molecular ions and antiprotonic helium. Phys. Rev. Lett..

[8] Hori M (2011). Two-photon laser spectroscopy of antiprotonic helium and the antiproton-to-electron mass ratio. Nature.

[9] Hori M (2016). Buffer-gas cooling of antiprotonic helium to 1.5 to 1.7 K, and antiproton-to-electron mass ratio. Science.

[10] Abe K (2012). Measurement of the cosmic-ray antiproton spectrum at solar minimum with a long-duration balloon flight over Antarctica. Phys. Rev. Lett..

[11] Aguilar M (2016). Antiproton flux, antiproton-to-proton flux ratio, and properties of elementary particle fluxes in primary cosmic rays measured with the Alpha Magnetic Spectrometer on the International Space Station. Phys. Rev. Lett..

[12] Aramaki T (2016). Antideuteron sensitivity for the GAPS experiment. Astropart. Phys..

[13] Ahmadi M (2018). Characterization of the 1S–2S transition in antihydrogen. Nature.

[14] Baker C (2021). Laser cooling of antihydrogen atoms. Nature.

[15] DiSciacca J (2013). One-particle measurement of the antiproton magnetic moment. Phys. Rev. Lett..

[16] Ulmer S (2015). High-precision comparison of the antiproton-to-proton charge-to-mass ratio. Nature.

[17] Bakalov D, Jeziorski B, Korona T, Szalewicz K, Tchoukova E (2000). Density shift and broadening of transition lines in antiprotonic helium. Phys. Rev. Lett..

[18] Bakalov D (2012). Density shift and broadening of dipole transitions in antiprotonic helium. Hyperfine Interact..

[19] Badertscher A (1997). Experimental determination of the kinetic energy distribution of *π*^−^*p* atoms in liquid hydrogen. Phys. Lett. B.

[20] Siems T (2000). First direct observation of Coulomb explosion during the formation of exotic atoms. Phys. Rev. Lett..

[21] Jensen TS, Markushin VE (2002). Collisional deexcitation of exotic hydrogen atoms in highly excited states. Eur. Phys. J. D.

[22] Fetkovich JG, McKenzie J, Riley BR, Wang I-T (1975). Measurement of the cascade time of Σ^−^ in liquid helium. Nucl. Phys. A.

[23] Glyde, H. R., Azuah, R. T. & Stirling, W. G. Condensate, momentum distribution, and final-state effects in liquid ^4^He. *Phys. Rev. B* **62**, 14337–14349 (2000).

[24] Zheng-Johansson, J. X. & Johansson, P.-I. *The Microscopic Theory of Superfluid He II and its QCE Superfluidity Mechanism Applied to Superconductors: Theory of Condensed Matter Expounded Through the System He II* (Nova Science Publishers, 2004).

[25] Diallo SO (2014). Evidence for a common physical origin of the Landau and BEC theories of superfluidity. Phys. Rev. Lett..

[26] Dmowski W (2017). Observation of dynamic atom–atom correlation in liquid helium in real space. Nat. Commun..

[27] Lemeshko M (2017). Quasiparticle approach to molecules interacting with quantum solvents. Phys. Rev. Lett..

[28] Ishikawa K (1997). Laser spectroscopy of thulium atoms implanted in liquid and solid ^4^He. Phys. Rev. B.

[29] Hui Q, Takami M (2000). Phonon bands associated with the inner-shell electronic absorption lines of Eu atoms in bulk liquid helium. J. Low Temp. Phys..

[30] Moroshkin P, Lebedev V, Weis A (2011). Phonon generation in condensed ^4^He by laser-excited atomic bubbles. Europhys. Lett..

[31] Moroshkin P, Borel A, Kono K (2018). Laser spectroscopy of phonons and rotons in superfluid helium doped with Dy atoms. Phys. Rev. B.

[32] Moroshkin P, Kono K (2019). Zero-phonon lines in the spectra of dysprosium atoms in superfluid helium. Phys. Rev. B.

[33] Russell, J. E. Metastable states of *απ*^−^*e*^−^, *αK*^−^*e*^−^, and $$\alpha \bar{p}{e}^{-}$$ atoms. *Phys. Rev. Lett.* **23**, 63–64 (1969).

[34] Fetkovich JG, Riley BR, Wang IT (1971). The atomic cascade of negative particles in liquid helium. Phys. Lett. B.

[35] Iwasaki M (1991). Discovery of antiproton trapping by long-lived metastable states in liquid helium. Phys. Rev. Lett..

[36] Hori M (2001). Sub-ppm laser spectroscopy of antiprotonic helium and a CPT-violation limit on the antiprotonic charge and mass. Phys. Rev. Lett..

[37] Bakalov D, Korobov V (1998). Hyperfine structure of antiprotonic helium energy levels. Phys. Rev. A.

[38] Korobov VI, Zhong Z-X, Tian Q-L (2015). Leading term of the $${\rm{He}}-\bar{p}{{\rm{He}}}^{+}$$ long-range interaction. Phys. Rev. A.

[39] Hizhnyakov V, Boltrushko V, Benedek G (2021). Thermal broadening of the zero-phonon line in superfluid helium. Phys. Rev. B.

[40] Hartmann M, Miller RE, Toennies JP, Vilesov A (1995). Rotationally resolved spectroscopy of SF_6_ in liquid helium clusters: a molecular probe of cluster temperature. Phys. Rev. Lett..

[41] Grebenev S, Toennies JP, Vilesov AF (1998). Superfluidity within a small helium-4 cluster: the microscopic Andronikashvili experiment. Science.

[42] Hori M, Aghai-Khozani H, Sótér A, Dax A, Barna D (2020). Laser spectroscopy of pionic helium atoms. Nature.

[43] Nozzoli, F., Dimiccoli, F., Iuppa, R., Riccia, E. & Zuccona, P. An helium calorimeter for antideuteron identification in cosmic rays. *In Proc. 37th International Cosmic Ray Conf. (ICRC2021)* (IUPAP, 2021).

[44] Donato, F., Maurin, D., Brun, P., Delahaye, T. & Salati, P. Constraints on WIMP dark matter from the high energy PAMELA $$\bar{p}/p$$ data. *Phys. Rev. Lett.* **102**, 071301 (2009). 10.1103/PhysRevLett.102.071301 19257657

[45] Cuoco A, Krämer M, Korsmeier M (2017). Novel dark matter constraints from antiprotons in light of AMS-02. Phys. Rev. Lett..

[46] Turner MS (1982). Could primordial black holes be the source of the cosmic ray antiprotons?. Nature.

[47] Maki K, Mitsui T, Orito S (1996). Local flux of low-energy antiprotons from evaporating primordial black holes. Phys. Rev. Lett..

[48] Barrau A (2002). Antiprotons from primordial black holes. Astron. Astrophys..

[49] Fujimoto R (2017). Performance of the helium dewar and the cryocoolers of the Hitomi soft x-ray spectrometer. J. Astron. Telesc. Instrum. Syst..

[50] Gehrz RD (2007). The NASA Spitzer Space Telescope. Rev. Sci. Instrum..

[51] Hori M (2002). Primary populations of metastable antiprotonic ^4^He and ^3^He atoms. Phys. Rev. Lett..

[52] Filippov YP, Miklyaev VM (2019). A comparison of two kinds of TVO cryogenic temperature sensors. Cryogenics.

[53] Arp, V. D., McCarty, R. D. & Friend, D. G. Thermophysical Properties of Helium-4 from 0.8 to 1500 K with Pressures to 2000 MPa. Technical note 1334 (NIST, 1998).

[54] Preston-Thomas H (1990). The International Temperature Scale of 1990 (ITS-90). Metrologia.

[55] McCarty, R. D. & Arp, V. D. A new wide range equation of state for helium. In Advances in Cryogenic Engineering (ed. Fast, R. W.) 1465–1475 (Springer, 1990).

[56] Donnelly RJ, Barenghi CF (1998). The observed properties of liquid helium at the saturated vapor pressure. J. Phys. Chem. Ref. Data.

[57] Agostinelli S (2003). GEANT4—a simulation toolkit. Nucl. Instrum. Methods Phys. Res. A.

[58] Bylinsky, Y., Lombardi, A. M. & Pirkl, W. RFQD - a decelerating radio frequency quadrupole for the CERN antiproton facility. In *Proc. 20th Intl. Linear Accelerator Conf.* (ed. Chao, A. W.) 554–556 (SLAC, 2000)

[59] Bielert ER, Verweij AP, ten Kate HHJ (2013). Implementation of the superfluid helium phase transition using finite element modeling: simulation of transient heat transfer and He-I/He-II phase front movement in cooling channels of superconducting magnets. Cryogenics.

[60] Bielert ER, ten Kate HHJ, Verweij AP (2015). A structured approach to analyze the influence of channel dimensions on heat extraction via superfluid helium. Phys. Proc..

[61] Hori M, Yamashita K, Hayano RS, Yamazaki T (2003). Analog Cherenkov detectors used in laser spectroscopy experiments on antiprotonic helium. Nucl. Instrum. Meth. A.

[62] Hori M (2005). Photocathode microwire monitor for nondestructive and highly sensitive spatial profile measurements of ultraviolet, x-ray, and charged particle beams. Rev. Sci. Instrum.

[63] Hori M, Dax A (2009). Chirp-corrected, nanosecond Ti:Sapphire laser with 6 MHz linewidth for spectroscopy of antiprotonic helium. Opt. Lett..

[64] Diddams SA, Vahala K, Udem T (2020). Optical frequency combs: coherently uniting the electromagnetic spectrum. Science.

